# Playing off the curve - testing quantitative predictions of skill acquisition theories in development of chess performance

**DOI:** 10.3389/fpsyg.2014.00923

**Published:** 2014-08-22

**Authors:** Robert Gaschler, Johanna Progscha, Kieran Smallbone, Nilam Ram, Merim Bilalić

**Affiliations:** ^1^Universität Koblenz-Landau, Landau, Germany and Interdisciplinary Research Laboratory Image, Knowledge, Gestaltung at Humboldt-UniversitätBerlin, Germany; ^2^Department of Psychology, Humboldt-UniversitätBerlin, Berlin, Germany; ^3^School of Computer Science, University of ManchesterManchester, UK; ^4^College of Health and Human Development, Human Development and Family Studies, Pennsylvania State UniversityUniversity Park, PA, USA; ^5^Alpen-Adria-Universität Klagenfurt, Institut für Psychologie, Abteilung für Allgemeine Psychologie und KognitionsforschungKlagenfurt, Austria

**Keywords:** learning curves, skill acquisition, expertise, chess, development

## Abstract

Learning curves have been proposed as an adequate description of learning processes, no matter whether the processes manifest within minutes or across years. Different mechanisms underlying skill acquisition can lead to differences in the shape of learning curves. In the current study, we analyze the tournament performance data of 1383 chess players who begin competing at young age and play tournaments for at least 10 years. We analyze the performance development with the goal to test the adequacy of learning curves, and the skill acquisition theories they are based on, for describing and predicting expertise acquisition. On the one hand, we show that the skill acquisition theories implying a negative exponential learning curve do a better job in both describing early performance gains and predicting later trajectories of chess performance than those theories implying a power function learning curve. On the other hand, the learning curves of a large proportion of players show systematic qualitative deviations from the predictions of either type of skill acquisition theory. While skill acquisition theories predict larger performance gains in early years and smaller gains in later years, a substantial number of players begin to show substantial improvements with a delay of several years (and no improvement in the first years), deviations not fully accounted for by quantity of practice. The current work adds to the debate on how learning processes on a small time scale combine to large-scale changes.

## Introduction

Anderson (2002) drew attention to the problem of time scales in psychology with the programmatic article *Spanning Seven Orders of Magnitude*. On the one hand, acquisition of expertise is known to takes years (e.g., Ericsson et al., 1993). On the other hand, expertise research has a strong basis in cognitive psychology paradigms wherein a large repertoire of laboratory tasks are used to understand and chart changes in potential subcomponents of expertise acquisition over minutes or hours. This includes component skills such as verifying and storing chess patterns (Gobet and Simon, 1996a,b,c, 1998; Campitelli et al., 2005, 2007; Bilalić et al., 2009a), learning to discard irrelevant perceptual features from processing (e.g., Gaschler and Frensch, 2007; Reingold and Sheridan, 2011) or overcoming dysfunctional bindings of knowledge structures (e.g., Bilalić et al., 2008a,b). Anderson suggested that while meaningful educational outcomes take at least tens of hours to achieve, those outcomes can be traced back to operations of attention and learning episodes at the millisecond level. He went beyond offering the perspective that expertise acquisition should *in principle* be reducible to small scale learning episodes. Rather, Anderson suggested that the problem of linking domains of (a) laboratory cognitive psychology/neurocognitive research and (b) educational/developmental science should be tractable, because small scale learning episodes would sum up to large scale developmental/educational changes of the same functional form. Increases in overall performance as well as increases in efficiency of components (e.g., keystrokes, eye movements and fact retrieval) over time are well described by the power function (see also Lee and Anderson, 2001). Power functions of improvements in simple components add up to a power-function improvement at the large scale. Scalability across time-scales would offer straightforward linking of change taking place within minutes to change taking place over years.

**Figure 1:**
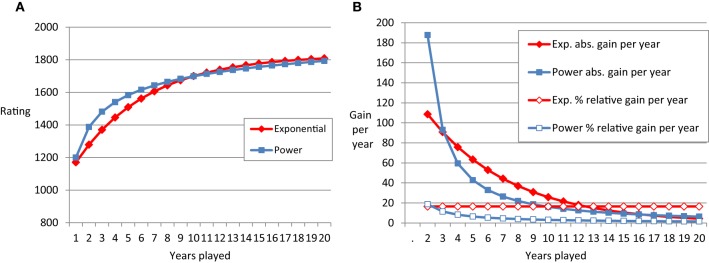
**(A)** Schematic plot of the power function (blue lines) and negative exponential function (red lines) over 20 time points. **(B)** Shows the absolute differences in performance from one time point to the next (filled symbols) and the relative learning rate (empty symbols).

The power function (as well as the negative exponential function, see Table 1 and Figure 1) describes negatively accelerated change of performance with practice. Early in practice, the absolute improvement in performance per unit of time invested is large. Later on, the improvement per unit of time diminishes. Apart from improvements in hour-long laboratory learning tasks, the power function has been used to describe motor skills in individuals differing in amount of practice on the scale of years (e.g., up to 7 years of cigar-rolling in Crossman, 1959, see Newell et al., 2001 for an overview). Description of practice gains with the power function are widespread in the literature (Newell and Rosenbloom, 1981; Kramer et al., 1991; Lee and Anderson, 2001; Anderson, 2002) and consistent with prominent models of skilled performance such as ACT-R (Anderson, 1982) or the instance model of automatization (Logan, 1988, 1992).

**Table 1:** **Formulas of negative exponential and power function and the example parameters used for Figure 1**.

**Formula**		**Negative exponential function**	**Power function**
		Rating = A − B * exp(−C * T)	Rating = A − B* T** − C
Parameter descriptions and numerical examples from Figure 1	A = asymptote	1830	2200
	B = difference between asymptote and initial performance	790	1000
	C = curvature	0.18	0.3
	T = time	Year 1–20	Year 1–20

However, the reason for the dominance of the power function in describing the functional form of describing practice has been debated in the literature on skill acquisition. Heathcote et al. (2000) see also Haider and Frensch (2002) argued that the analysis of averaged data favors the power over the exponential function as a statistical artifact. They suggested computation of power and exponential curves with non-aggregated data, separately for each participant. They found an advantage of the negative exponential function over the power function in 33 of 40 different re-analyzed data sets with an average improvement in fit of 17%. Note that success of a mathematical model in fitting data better than a competitor model might not mean that it provides a more concise description. Potentially, one mathematical model is more flexible than the other, and better able to accommodate systematic as well as chance features in the data. Thus, further credence is lent to a model by accurate prediction rather than fitting (i.e., without any further parameter adjustments; cf. Roberts and Pashler, 2000; Pitt et al., 2002; Wagenmakers, 2003; Marewski and Olsson, 2009).

It is worthwhile considering the exact shape of the learning curve to predict future performance. Furthermore, the differences between exponential and power function are linked to assumptions in theories of skill acquisition (see below). Figure 1 represents schematic examples of learning curves and derivatives. The left panel depicts a power function and an exponential function that start at the same level in the first year of chess tournament participation and approach similar levels in year 20 of tournament participation. The power function shows especially strong performance gains in the first years. For instance, the gain in rating points (e.g., Elo, 1978) in year one is about double the size of the gain in year two. Year two still yields considerably more performance gain as compared to year three, and so on and so forth. Absolute gain per year is depicted in the right panel. It is decreasing for both, the power and the exponential function. The qualitative difference between the two types of learning curves becomes most obvious when considering the relative learning rate (RLR). This rate is decreasing for the power function, but remains constant for the exponential function. In our example, the exponential function has a relative learning rate of about 20%. In each year, the players gain about 20% of the ELO points they have not gained yet. If someone starts with 1000 and will end up with 1500 points (see Method for an explanation of the scale used in chess), this would mean a gain of 100 points for the first year and 80 points in the second year (20% of 1500 − (1000 + 100) = 80 points).

One qualitative aspect of learning curves is that they represent the diminishing absolute payoff of practice-investment. Exponential practice functions can be derived from a narrow set of assumptions. As Heathcote et al. (2000) explained one needs only to assume that learning is proportional to the time taken to execute the component in case of a continuous mechanism. First, a component that takes longer to execute presents more opportunity for learning. Second, as learning proceeds, the time to execute the component decreases. Therefore, the absolute learning rate decreases, resulting in exponential learning. Similarly, for discrete mechanisms, such as chunking, exponential learning can be explained by a reduction in learning opportunity. As responses are produced by larger and larger chunks, fewer opportunities for further composition are available. Time-demanding control is no longer necessary for small steps but only for scheduling sets consisting of fixed series of small patterns. Naturally, the opportunities for compilation of small single knowledge units into larger ones reduce, as more and more patterns are already chunked.

Additional theoretical assumptions are needed to accommodate a decreasing RLR. For instance, Newell and Rosenbloom (1981) see also Anderson (2002) assumed that chunks are acquired hierarchically and that every time a larger chunk is practiced, this entails practice of its smaller components. Thus, by practicing a knowledge unit consisting of sub-units, the sub-units and the overall pattern are fine-tuned and strengthened. Furthermore, at least in combinatorial environments, acquisition proceeds ordered by chunk span. No larger span chunk is acquired until all chunks of smaller span have been acquired.

The above research suggests that one or the other simple learning function might be adequate to describe improvements over long time intervals (cf. Howard, 2014). Functions known from work on short-term skill acquisition should be relevant to describe long-term expertise acquisition. We take chess as an example to explore this perspective. First, longitudinal data spanning years of practice are available. Second, theories on expertise in chess can be taken to suggest that scalability between small scale learning and large scale expertise acquisition should be especially likely to hold in this domain. Expertise development in chess might predominantly be based on cumulatively storing more and more patterns of chess positions (Chase and Simon, 1973; Gobet and Simon, 1996b). Spatial (Waters et al., 2002; Connors and Campitelli, 2014; Leone et al., 2014) and perceptual capabilities are deemed crucial (Charness et al., 2001; Reingold et al., 2001; Bilalić et al., 2008a,b; Kiesel et al., 2009; Bilalić et al., 2010; Bilalić and McLeod, 2014). This suggests that attentional and learning episodes taking place at the time scale of milliseconds might together lead to expertise acquisition. This in turn would make it likely that expertise acquisition can be described by the learning function exhibited during learning episodes that take place within a single laboratory session.

In order to explore the potential of this conjecture in the current study, we provide a descriptive analysis of the development of chess performance in German players who start playing chess at an early age and continue with the activity for at least 10 years. Relevant for theoretical as well as practical purposes, the time courses of expertise acquisition could thus potentially be predicted. Based on the shape of the curve of improvements during the first years of expertise acquisition, one might be able to predict the time course of improvements over the years of practice to come (Ericsson et al., 1993; Charness et al., 2005).

## Methods

### Database

We used archival data of the population of German players recorded by the German chess federation (Deutscher Schachbund) from 1989 to 2007. Data were kindly provided by the federation and analyzed in line with guidelines of the ethics review board at Humboldt-Universität, Berlin. With over 3000 rated tournaments in a year, the German chess federation is one of the largest and the best-organized national chess federations in the world. Given that almost all German tournaments are rated, including events such as club championships, the entire playing careers of all competitive and most hobby players in Germany are tracked in detail. This is particularly important because we wanted to capture the very first stage of chess skill acquisition by focusing on the very young chess players who just started to play chess. The German database provides a perfect opportunity to study the initial stages of skill acquisition because even school tournaments are recorded.

### The measure—chess rating

Besides precise records of players, the German federation's database and chess databases in general use an interval scale, the Elo rating, for measuring skill level. Every player has an Elo rating that is obtained on the basis of their results against other players of known rating (see Elo, 1978). Average players are assumed to have rating of 1500 Elo points, experts over 2000 points, grandmaster, the best players, over 2500. Beginners usually start at around 800 Elo points. The German database uses the same system but labels the rating as Deutsche Wertzahl (DWZ), which is highly correlated (*r* > 0.90) with the international Elo rating (Bilalić et al., 2009b).

### Selection criteria and grouping of data analyzed

The German chess federation database contains records of over 124,000 players and the average rating of these players is 1387 points with standard deviation of 389 points. For all practical purposes, the database contains the entirety of the population of tournament chess observations in Germany (for more information about the database, see Bilalić et al., 2009b; Vaci et al., 2014). With interest in expertise development (rather than maintenance), we used the subset of data from all players who entered the database between age 6 and 20. This population consisted of 1383 players that played competitive chess for at least 10 years. All players took part in tournaments in each of the 10 years. To be sure that the initial observation was indeed first entry into competitive chess, we excluded players who were already listed in the first year the federation started tracking players. For the players starting young, there should have been little opportunity for expertise acquisition prior to taking part in tournaments covered by the database. To track this issue, we split the sample into age-groups (see Table 2 gender and age as well as for means and standard deviations of games played, rating reached by year 10, change in rating between year 1 and 10, and change in rating per game played).

**Table 2:** **Sample characteristics and summary statistics on games played and ratings**.

**Age**	**N (females)**	**Mean age (*SD*)**	**Mean total number of games (*SD*)**	**Mean rating in Year 10 (*SD*)**	**Mean rating change from Year 1–10 (*SD*)**	**Mean rating change per games played (*SD*)**
6–9	173 (24)	8.3 (0.9)	359.9 (196)	1705.1 (308.2)	894 (368.6)	3.21 (1.72)
10–13	689 (72)	11.6 (1.1)	239 (135,8)	1668.8 (268.9)	640.7 (294.9)	3.52 (2.15)
14–17	414 (22)	15.2 (1.1)	201.9 (116.1)	1660 (231.6)	457.4 (235.5)	3.04 (1.9)
18–20	107 (3)	18.8 (0.8)	180 (126.7)	1582.5 (200.6)	270.0 (201.7)	1.96 (1.5)

Note that since we are working with the entirety of tournament chess performances in Germany since 1989, we provide description of the entire population of interest—chess players that played competitive chess in Germany for at least 10 years (means, standard deviations, correlations that allow for an estimation of effect sizes). Generalization of findings, beyond the internal predictions, will have to be based on replications with other or future databases (see e.g., Asendorpf et al., 2013).

### Predicting and fitting with the power and exponential function

Fits were derived with constrained optimization, requiring the A and B parameters to take sensible values (0 < B < A < 3000) using the MATLAB Curve Fitting Toolbox. For each participant we compared estimated and observed ratings and determined whether the power function or the exponential function led to a smaller squared deviation. For predictions, we only used the data of the first 5 years to extract the parameters of the power function and the exponential function. Then we used these parameters to extrapolate the predicted ratings for the next years (at least 5—each person in the sample had database entries for a minimum of 10 years). The predicted values were then compared to the ratings actually achieved. For instance, for a given participant who played for 10 years, we took the performance in the first five, acting as if the trajectory data of the next years were not yet available. The power function and the exponential function were fit to the data of the first 5 years in order to obtain the parameter values exemplified in Table 1. Next we used these values in order to extrapolate for the coming years of tournament participation. These predicted values were than compared to the actual ratings obtained. For each participant we could thus compare the root mean square error (RMSE) between power function-based prediction and prediction based on the exponential function.

## Results

### Descriptive statistics

Table 2 indicates that our sample was predominantly male. Participants starting to play tournaments younger accumulated more games as compared to those starting at an older age. For instance, the 6 to 9 year olds played twice as many games than the 18 to 20 year olds. The rating reached by Year 10 was similar across age groups. Yet, this implied a much stronger improvement compared to Year 1 for the players starting young rather than old. For instance, the youngest group showed trifold the increase of the oldest group. The increase in rating relative to the number of games played was similar across age groups (with the players starting oldest, who showed a reduced gain per games played).

### Exponential better than power function in predictions and fits

**Figure 2:**
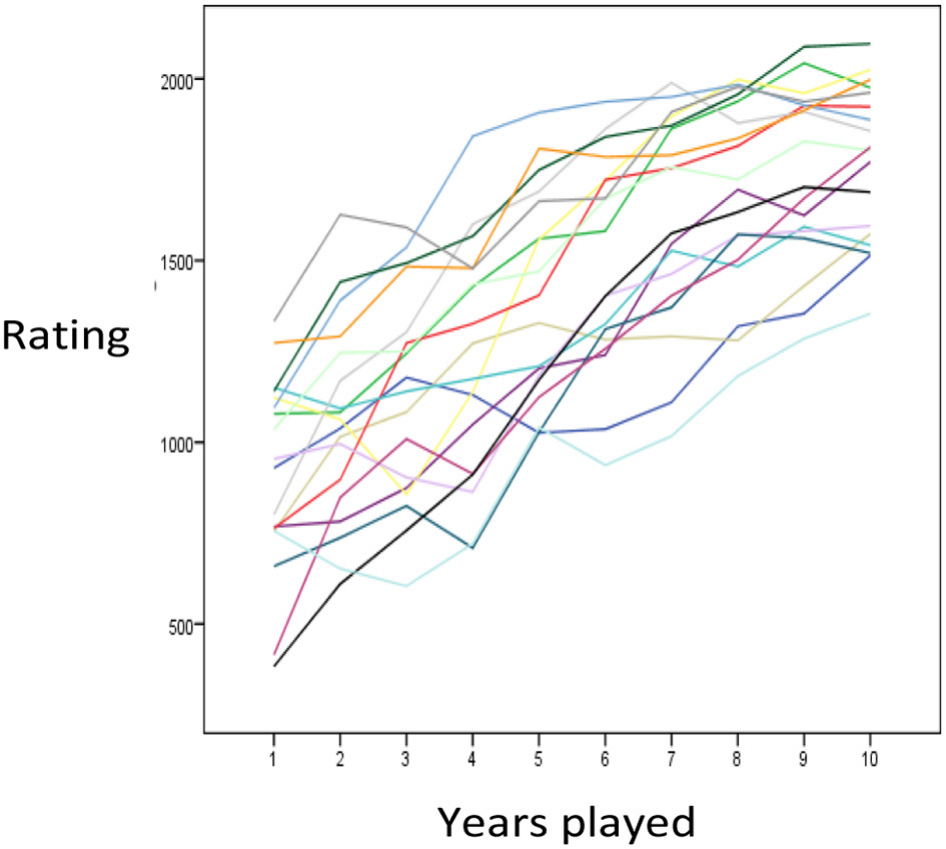
**A random sample of individual time courses**.

Figure 2 presents a random subset of individual time courses. Despite fluctuations from one year to the next, participants generally showed increases in skill, as measured by Elo, over years of chess played. Some participants showed large gains especially in their first years. In order to systematize such observations, we tested the capability of the power function and the exponential function to fit and predict the observed trajectories. Prediction is interesting for practical purposes as we can infer the skill level someone will have after ten years of activity based on the pattern of performance in their first years. On the other hand, prediction circumvents methodological problems inherent in curve fitting. For instance, one mathematical function might fit better than another, because it is flexible enough to mimic the competitor.

**Figure 3:**
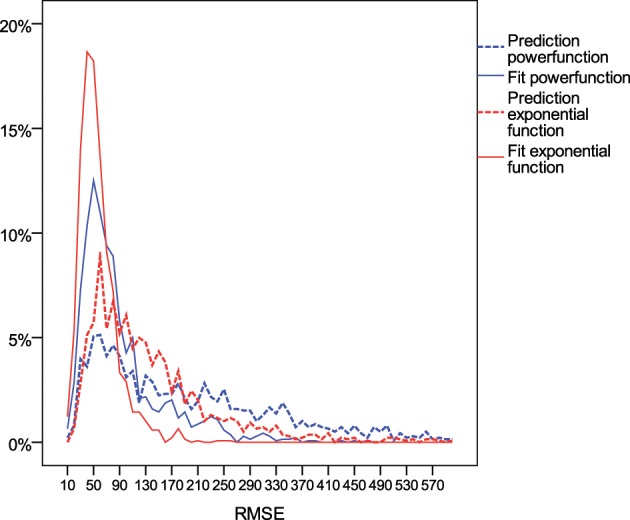
**Distribution of root mean square error (RMSE) of rating points of the power function (blue) and exponential function (red) in fitting the time course of the first 5 years (filled lines) vs. predicting the time course for the next years based on the parameters derived from fitting the first years (dotted lines)**.

Across individuals from all age groups, the exponential function provided better prediction and fit to the data than the power function (Table 3). The average RMSE and its standard deviation were smaller for the exponential function than for the power function (with exception of the prediction among those starting chess at ages 18–20). For 88% of the players, the exponential function was better in fitting the first 5 years the skill acquisition process, and for 62% it was better in predicting the skill level in later years. As shown in Figure 3, the distribution of RMSE values was heavily left-skewed. For a substantial proportion of participants neither the exponential nor the power function provided an account of the dynamics of individuals' skill development.

**Table 3:** **Average and standard deviation of RMSE per age group**.

	**Prediction**		**Fitting**	
**Age**	**Power**	**Exponential**	**Power**	**Exponential**
6–9	381.3 (179.9)	197.5 (127.4)	143.6 (88.7)	83 (37.8)
10–13	225.4 (135.6)	149.8 (99.3)	96 (62.8)	60.3 (28.1)
14–17	115.4 (84)	104.1 (63.3)	69.9 (45.9)	45.4 (20.4)
18–20	75.1 (48.1)	87.8 (56.7)	51.8 (30.5)	39 (19.6)

### Increasing gains in participants starting young

**Figure 4:**
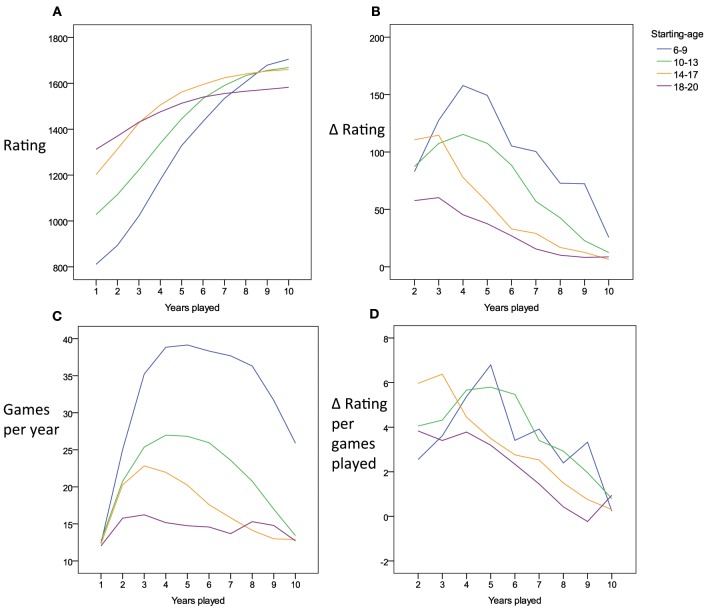
**(A)** Charts the average time course in tournament performance for players starting at 6–20 years of age. **(B)** Shows the time course of the gain in performance from one year to the next for the different starting-age groups. In **(C)** the time course of number of tournament games played per year is charted for the different starting-age groups. **(D)** Shows the time course of the improvement in tournament performance from year to year relative to the number of tournament games played in the respective year.

We grouped players by the age they started to play tournaments in order to explore reasons for the substantial problems in fit and prediction encountered with both the power and exponential functions. Potentially, players starting tournament participation at older ages might have profited from substantial opportunities to practice chess before they entered our window of analysis. Thus, the expected learning gains might manifest more readily in those players that started at younger ages. Figure 4A indeed shows that players entering tournament chess at older ages demonstrate higher skill levels by the end of their first year, while players entering at younger ages, start at lower levels. Most notably, however, the shape of the improvements deviates systematically from the patterns of change that would be expected based on either the exponential or the power function. Both learning functions predict that participants should show a higher absolute gain in rating points from the first to the second year compared to the gain from the second to the third year, which in turn should yield a higher gain as compared to the change from the third to the fourth year, and so on and so forth. Among the subset of players starting young, however, the contrary seemed to be the case (see also Figure 4B for difference values). Over the first years of tournament participation, the absolute amount of gain per year *increased* rather than decreased. The deviation from the expected learning curve might be related to year-to-year variations in practice. For example, the players starting young may, at first, participate in very few tournaments, and then, in the next few year, increase in the number of tournament games they take part in. This is indeed the case (Figure 4C). Therefore, it is conceivable that the amount of practice which increases over the years accounts for the dynamics of the skill increase—only once the players starting young take part in more and more games, their skill might start to increase in the manner predicted by the learning curves. As we do not possess any further data on changes in the amount of practice per year (i.e., off tournament practice), we cannot conclusively judge this account. However, at least we can state that the increase in the number of tournament games played cannot fully account for the dynamics. As shown in Figure 4D, the change in rating per year per number of tournament games played also shows an increase over the first years for players starting young.

### Fluctuations in games played per year relate to misfit with power function

It is conceivable that the misfit and inaccurate prediction of the power and the exponential functions are related to variability in the number of games played per year. While we only examined the subset of players who played in tournaments in each of the 10 years tracked, the number of games per year might have fluctuated. We computed the within-person (intraindividual) standard deviation of games played per year, assuming that fits should be optimal if the number of games a player takes part in does not change over the years. This index is equivalent to computing the deviation from a zero-slope line in numbers of games played. Table 4 shows the Spearman rank order correlation of intraindividual variability in number of games played per year with the RMSE obtained from fitting and predicting ratings based on the power function and the exponential function. The correlations suggest that larger intraindividual variability in number of games played per year was weakly but consistently related to worse fit in case of the power function (while the pattern was less consistent for the exponential function). A similar pattern was observed when correlating the overall number of games played to accuracy in prediction and fitting. Participants who played more games showed worse fits compared to participants who played less games. This was likely the case, because the number of games played over the ten years (a count variable) was closely linked to the intraindividual variability in games per year (Spearman correlations ranging between 0.84 and 0.88 across the four age groups).

**Table 4:** **Spearman rank order correlations of (a) indices of regularity in numbers of games played per year with (b) the fitting and prediction error**.

	**Age**	**Prediction**		**Fitting**	
		**Power**	**Exponential**	**Power**	**Exponential**
*SD* of games per year	6–9	0.13	−0.22	0.29	−0.05
	10–13	0.14	−0.16	0.45	0.20
	14–17	0.22	0.02	0.36	0.24
	18–20	0.18	0.20	0.47	0.44
Overall number of games	6–9	0.22	−0.30	0.37	−0.01
	10–13	0.21	−0.14	0.50	0.21
	14–17	0.24	−0.02	0.39	0.22
	18–20	0.18	0.23	0.56	0.48
Prototypical profile in games per year	6–9	−0.03	−0.36	0.21	−0.02
	10–13	−0.06	−0.17	0.06	−0.06
	14–17	0.04	−0.08	0.08	0.05
	18–20	0.22	−0.01	0.23	0.22

Figure 4C suggests that variability in number of games played per year is not purely random. Instead it can be based on an ordered pattern (inverted U-shape). Separately for each age group, we took the average profile in number of games played per year (displayed in Figure 4C) as a prototypical pattern. Then, we determined for each participant the profile correlation between his/her pattern of numbers of games played with the average pattern of the respective age group. Our analyses suggested that there was substantial variability, with some participants following the pattern represented in the group mean and others deviating from it. Median within-person correlations per age group were *r* = 0.58, 0.5, 0.47, and 0.19. The percentage of individuals showing a negative correlation with the prototypical pattern was 9.8, 15.2, 19.6, and 31.8%. However, as suggested by Table 4, the extent to which the dynamics of an individual's number of games played per year was represented by the average pattern of the age group was not systematically related to the accuracy in power function or exponential function fits and predictions.

### Off-the-curve patterns in 2/3^rds^ of the sample

**Figure 5:**
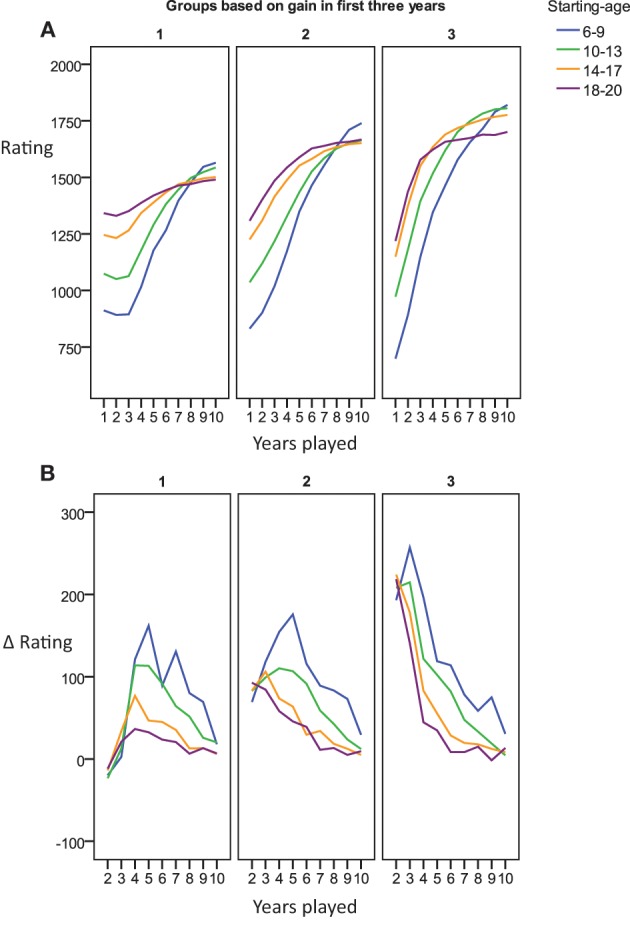
**(A)** Charts the average time course in tournament performance for players starting at 6–20 years of age grouped by improvement over the first three years. **(B)** Shows the change in ELO per year.

We sought to provide descriptive data on the number of participants who deviated from the predictions of the learning curves by showing smaller rather than larger rating gains during their early as compared to their later years of tournament participation. For this we sorted individuals into tertiles based on the total gains achieved during the first 3 years (lowest, medium, and highest rating gains). As shown in Figure 5A, the third of players with the lowest gains even showed small decreases in rating during the first years, while only the individuals with the largest gain yielded performance changes in line with the predictions by the learning curves (i.e., larger gain per year in early rather than late years, compare Figure 5B). Players that did not improve in their first three tournament years caught up to some extent in later years, but did not reach the same level by year 10 as those players with a steep increase early on. Thus, irrespective of complex dynamics of the shape of the performance curve, the first years do seem to offer a proxy for predicting the level a player will eventually reach.

### Cohort differences

**Figure 6:**
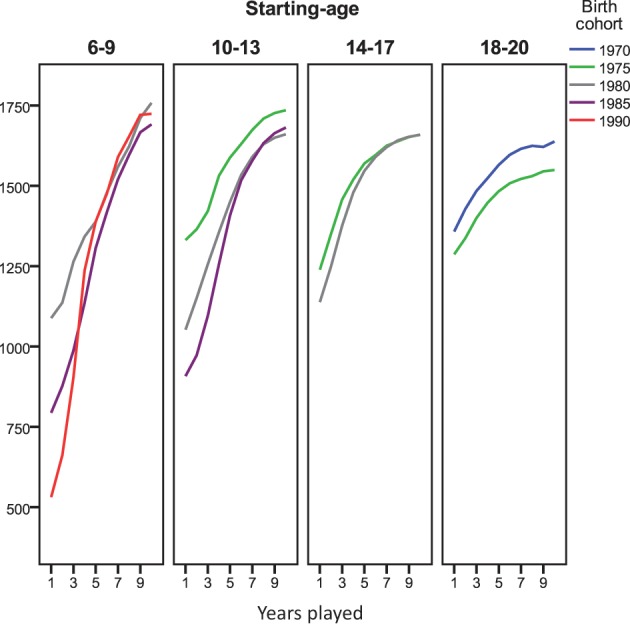
**Average time course in tournament performance for players starting at 6–20 years of age grouped by birth cohort (1970–1990)**. Due to small n (11) we dropped the 14–17 year old starters born between 1970 and 1975.

There have been many changes in resources available for chess players since 1989. We analyzed the time course in development of chess ratings separately for different cohorts in order to explore whether deviations from the pattern predicted by the learning curves varied in relation to the historical period that a chess career was started. Deviations from the learning curve were not accounted for by cohort. Rather, for all 5-year cohorts from 1970 to 1990 and age-groups displayed in Figure 6, the increase in rating during the first years of performance was linear or positively accelerated. The pattern of negative acceleration (larger gains in earlier as compared to later years, compatible with the learning curves) was not observed.

Age-groups and cohorts differed more with respect to the rating level they started out with (i.e., reached by end of their first year) than with respect to their level of performance in Year 10. As already observable in Figure 5A, people starting to play tournaments at younger age, started out at a lower level. In addition, Figure 6 shows that later cohorts started at lower levels. This might be taken to suggest that players starting young in late cohorts are the best candidates to track trajectories in chess performance based on tournament ratings, while ratings of players starting older and earlier cohorts might be shaped more strongly by off-tournament practice.

### Gain in rating from games played

**Figure 7:**
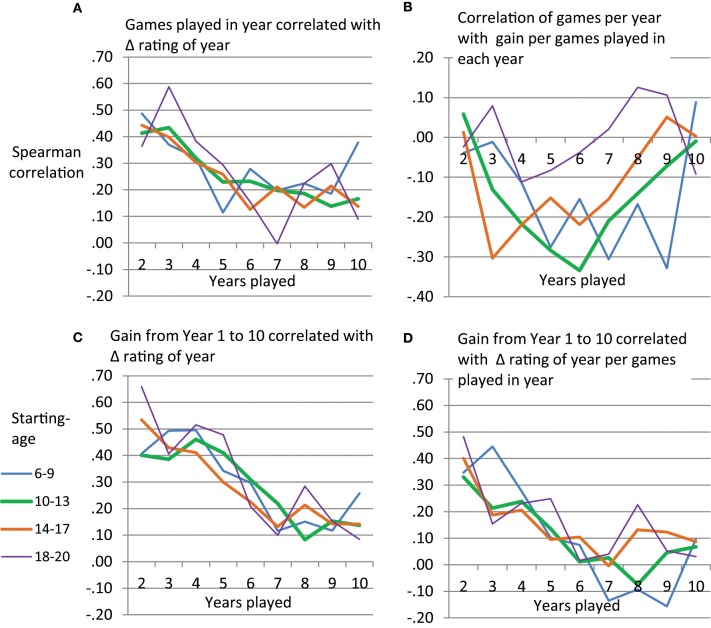
**Spearman rank order correlations per age group between performance variables taken from single years and rating gain per year (A,B) or overall rating gain (C,D)**.

The above analyses suggest that the success of the power function and the exponential function in predicting development of chess performance might be rather limited due to quantitative and qualitative misfit. Furthermore, the number of tournament games played seemed to be linked to deviations from the learning curve. Therefore, we sought to describe the extent to which early vs. late years in playing tournament chess are related to gain in rating as well as performance level reached by Year 10. For this we used games played per year and gain in rating per year. We applied Spearman rank order correlations separately for each age group and year of tournament participation. Figure 7A shows that number of games played per year is related to between-person differences in gain in rating. Participants playing more tournament games in a year tend to show a larger increase in rating compared to those playing less games. This holds consistently across age-groups and especially so for early years of tournament participation. However, diminishing returns seem to be observable with respect to the extent to which more tournament games can lead to an increase in rating. Figure 7B shows that the relationship between (a) games played per year and (b) gain per games played per year can become negative. Thus, overall it does not seem to be the case that playing more tournament games can lead to an increase in efficiency in taking gains in rating from a tournament game. For instance, those players starting tournament participation at age 10–13 who played more tournament games, seemed to show a reduced gain in rating per tournament game played in their middle years.

The gain in rating that players show from Year 1 to Year 10 can be predicted by gain in rating per year in early years of tournament participation. As depicted in Figure 7C, gain in later years is less predictive of the overall increase in rating. While the power and the exponential function would have predicted that we can observe large gains in rating in early years, we thus, somewhat analogously, observer a larger predictive power of between-person differences in early as compared to late years of chess tournament participation. Apart from the gain per year, also the gain per year relative to the number of games played per year could be used to predict the overall increase in rating between Year 1 and 10 (Figure 7D). Participants who, during the first years of tournament participation, efficiently increased their rating per games played, ended up at a higher performance level than those, who did not show a large gain per games played during early years.

### Selective attrition

Finally, we checked for selective attrition. While in our main analyses we only used 10 years of subsequent tournament participation, some participants provided records for additional years (up to 19 years overall). Rank order correlations indicated that the number of overall years of tournament participation per age group was neither systematically related to gain between Year 1 and Year 10, nor the gain in rating within the first 3 years (*r*s between −0.10 and 0.16).

## Discussion

In the current work we have explored the potential of the power- and the negative exponential learning functions to account for the development of chess performance measured in ratings based on tournament outcomes. In line with re-evaluations of the power law of practice (Heathcote et al., 2000; Haider and Frensch, 2002), we documented that the exponential function was better than the power function in fitting and predicting the time course of chess ratings over years of practice. However, a crucial aspect shared by both of these mathematical functions and the underlying theories of skill acquisition was not reflected in the data. While according to the power- as well as the negative exponential function players should achieve large absolute gains early in practice and small gains later in practice, this was not the case for many of the participants. Rather, many players started to show substantial improvements only *after* their first years of tournament participation. They were playing off the learning curves suggested by skill acquisition theories. If expertise acquisition is not well described by learning functions used to describe skill acquisition, the linking of underlying cognitive processes of attention and learning that proceed on time-scales measuring milliseconds to hours with learning processes that proceed on time-scales measured in years seems much less straightforward than one could have hoped for (i.e., Anderson, 2002).

Many players showed an acceleration of gain in rating in the first years of tournament participation, followed by a deceleration. Based on the power function and the exponential function we would have expected to only find the latter. Newell et al. (2001) suggested to mathematically and conceptually accommodate such findings by assuming a mixture of learning processes taking place on different time scales. Acceleration followed by deceleration could be captured by a sigmoid function that consists of two exponential components, a positive (acceleration) and a negative one (deceleration). Learning opportunities and efficiency in using them might increase during first years of tournament performance for many players, while in later years returns of investing in chess performance are diminishing. In line with this view, year-long trajectories of skill acquisition might be better understood from a perspective that takes lifespan-developmental and educational changes into account (Li and Freund, 2005). For instance, players starting to take part in tournaments at a young age are likely to promote changes in self-regulation strategies available (Lerner et al., 2001; Freund and Baltes, 2002) and acquire the potential to shape their social and learning environment. Their ability to learn about chess from (foreign language) media and options to travel to and communicate with other players will increase. Deliberate practice (cf. Ericsson et al., 1993) might require that young players develop skills to competently use of their motivational resources, by, for instance, scheduling work on skill acquisition such that as many of the activities as possible are intrinsically motivating (cf. Rheinberg and Engeser, 2010 as well as Christophel et al., 2014, for training of motivational competence). Underlining this challenge, Coughlan et al., 2014 reported that participants in the expert group of their study rated their practice as more effortful and less enjoyable compared to other participants. The experts were successful in improving performance, by predominantly practicing the skill they were weaker at. However, such gains in potential to learn might for many players no longer compensate for the physical and social changes faced during puberty (Marceau et al., 2011; Hollenstein and Lougheed, 2013), at the end of adolescence, during secondary education, family formation or labor force participation. Future research should thus try to simultaneously account for development in the individual, the opportunities provided by the environment (cf. Ram et al., 2013) and to model different trajectories in one framework (e.g., Grimm et al., 2010; Ram and Diehl, in press).

For skill acquisition mechanisms such as chunking, negative exponential learning can be explained by a reduction in learning opportunities (cf. Heathcote et al., 2000). The later in practice, the fewer chunks are yet to be learned. While a deceleration of learning should be observed late in practice, such an account does not preclude that strong increases in learning opportunities early in practice can lead to an acceleration of chunks acquired per time invested. It appears that, for at least some players, opportunities and efficiency in increasing chess performance are already fully present at the time they start to play tournaments. They start at the turning point of the sigmoid function. The “upper” negative exponential portion of the sigmoid is sufficient to describe their performance gains, which are large in their early years and then diminish as performance approaches the asymptote. For other players, both positive and negative exponential portions of the sigmoid function are needed to represent the dynamics of their chess performance over time. These players appear to be less saturated with respect to learning opportunities and efficiency when starting to take part in tournaments covered by the database. They thus first show an acceleration in rating gains per year, followed by the deceleration when approaching asymptote.

In line with these speculations, Howard (2014) reported an average trajectory of rating increases showing deceleration only for International Chess Federation (FIDE) players (rather than acceleration followed by deceleration). The shape of the curve reported by Howard matches the exponential curve from Figure 1A. Starting at an average of about 2200 points, the sample mean increased beyond 2500 points with practice. Different from the database used in the current study, the threshold to be listed in the FIDE database is high (cf. Vaci et al., 2014 for a discussion of problems implied by restriction of range in chess databases). Likely, players were already taking full advantage of opportunities to improve chess performance when entering the database so that an acceleration in rating gain with practice was no longer possible. Descriptive analyses suggest that the dynamic in rating improvement that players at the international level show with practice seems consistent with the negatively accelerated exponential function. As implied by the exponential function, the relative learning rate (RLR) estimated based on the average data published by Howard (2014) is constant. While the power function should lead to a decrease of RLR with practice (cf. Heathcote et al., 2000), the RLR is fluctuating around 20%. Focusing on the first half of practice in order to avoid inflation of RLR at the end of the practice curve, we obtained an *r* = 0.11 correlation of RLR with time point. Thus there was no hint toward a decrease.

Our correlational analyses suggest that interindividual variability in rating gain over the course of ten years of tournament participation can be predicted by between-person differences in performance during the first years. Even by taking data from single years, number of games played, rating points gained or rating points gained per games played, allow to predict overall gain at a moderate level. While the power and the exponential learning curve would suggest that the first years of practice should be important because of the large performance gains, we thus can somewhat analogously conclude that the first years are more important than later years for predicting between-person differences in performance level reached on the long run (cf. Ackerman and Woltz, 1994).

We focused on examining changes in rating with year of practice (rather than number of games played, cf. Howard, 2014). This allowed us to explore changes in rating gain and rating gain per games played with age and cohort. Yet, a direct comparison of the capability to capture performance change is lacking so far for the two potential time scales, (1) number of games played, (2) chronological time in years, as well as (3) a mixture of both scales. Several issues are worth considering when exploring the complexity of models needed to account for expertise acquisition over years, as compared to models of skill acquisition in hour-long laboratory sessions. In the lab, quantity and quality of practice per unit of time is usually well controlled. In skill acquisition processes outside the lab they might vary considerably over the years of practice an individual engages in. In addition, potential cohort differences should not be neglected (cf. Gobet et al., 2002; van Harreveld et al., 2007; Connors et al., 2011). Future work should consider how data on both, quantity of practice and quality of practice, can be used to explain the time course of chess skill development (cf. Baker et al., 2003; Charness et al., 2005; Gobet and Campitelli, 2007; Howard, 2014). Apart from obtaining data on the amount of off-tournament learning opportunities, available data sets could be used to gauge variability in specific aspects of the learning opportunities. For instance, taking part in tournaments with large spread in opponent strength might provide more opportunities for improvement as compared to tournaments with more homogenous competitors.

### Conflict of interest statement

The authors declare that the research was conducted in the absence of any commercial or financial relationships that could be construed as a potential conflict of interest.
